# It is not “Just Circumcision”

**DOI:** 10.12669/pjms.314.7689

**Published:** 2015

**Authors:** Ebru Yesildag

**Affiliations:** 1 Ebru Yesildag, MD. Associate Professor, Department of Pediatric Surgery, Namik Kemal University, Faculty of Medicine, Turkey

**Keywords:** Anomaly, Circumcision, Penis

## Abstract

**Objective:**

Circumcision is one of the most commonly performed operations during childhood. The procedure is often underestimated in areas where it is frequently executed due to social and religion-based indications. In fact it might be an opportunity to detect and to correct any existing penile anomaly. The aim of the study was to retrospectively evaluate the boys who were admitted to a hospital for circumcision and the outcome of the procedure.

**Methods:**

The boys who were brought to outpatient clinics for circumcision between 2009-2015, were retrospectively evaluated. The indications for hospital admission and the presence of associated penile anomalies were searched. All the boys were examined and operated by a single surgeon of the institution.

**Results:**

Nine hundred forty four boys were brought to pediatric surgery outpatient clinics in order to be circumcised. The operation was performed in 318 of them. The physical examination revealed penile anomalies in 29 of the 318 cases. The detected anomalies were webbed penis, penile torsion, hypospadias, chordee without hypospadias and meatal stenosis.

**Conclusions:**

The proper examination of the boys by a physician prior to circumcision provides the detection of penile anomalies which can be corrected at the same session. The arrangements for performing circumcision in hospitals by the medical staff should be favored. The misleading perception of underestimation of the procedure where it is ritually performed, should be corrected.

## INTRODUCTION

Circumcision is one of the most common surgical procedures performed during childhood.^1^,^2^ The indications for the operation might be recurrent urinary tract infections and pathologic phimosis but generally the social and religion-based factors play an important role in the family’s decision of circumcision.^1^ There is a wide spectrum of congenital penile abnormalities and these can be diagnosed with a proper physical examination prior to circumcision.^3^-^5^ They can not only be diagnosed but also be corrected at the same session with circumcision. The perception of circumcision as an operation should be imposed to the parents, especially in countries where the procedure is ritually performed, in order to be able to have it done in a hospital setting by the medical staff.

## METHODS

The boys who were admitted to outpatient clinics just for circumcision between July 2009 – January 2015, were retrospectively evaluated. The cases that asked for circumcision together with the correction of their main pathology such as undescended testicle or inguinal hernia, were not included into the study. All the boys were examined and operated by the single pediatric surgeon of the institution. The informed consent for the operation was obtained from the parents of each child in this series. The complaints of the cases, age at admission, seasonal variation of admission, incidence of penile anomalies were evaluated. Blood clotting tests were analyzed prior to operation. The surgical circumcision was performed mainly under general anesthesia, except those cases in whom neonatal circumcision under local anesthesia was applied. Dorsal penile nerve block with bupivacain was carried on in all patients. A dressing of a gauze containing a topical antibiotic cream was wrapped around the circumcised penis and baths were started 2 days after the operation. All patients were prescribed an oral analgesic at least for two days postoperatively.

## RESULTS

There were 944 boys who were admitted to Pediatric Surgery outpatient clinics for circumcision between July 2009 and January 2015. The mean age at admission was found to be 6.5 years (ranged between 2 days – 14 years). June was the leading month of admission for circumcision (27%, 251 cases) followed by May and January.

The 66% (623 / 944) of the cases did not have any complaints but just wanted circumcision for social / religion-based reasons. The 21% (197 / 944) of the boys were said to have difficulty in urination but they neither had urinary tract infection nor balanitis attacks in their history. The remaining 13% (124 / 944) of the patients had recurrent urinary tract infections (48 cases); more than one balanitis / posthitis attacks (39 cases) and prenatally detected urinary tract anomalies (37 cases). The reasons at admission for circumcision are presented in Table-I.

**Table-I:** The distribution of patients according to the reasons of admission for circumcision.

No. of cases	No. of cases	Complaints at admission
944 boys	623 (66%)	No complaints; Social & religious reasons
	197 (21%)	Difficulty in urination but no previous urinary tract infection or balanitis attacks
	124 (13%)	Urinary tract infection (48)
		>1 balanitis/posthitis attacks (39)
		Prenatally detected urinary anomaly (37)

Circumcision was performed in 318 (34%) patients of this series. General anesthesia was applied in 311 boys, while seven cases, all of whom were neonates, were operated under local anesthesia. The mean age at operation was 5 years ranging between 2 days to 11 years.

Penile anomalies consisting of webbed penis (n=14), chordee without hypospadias (n=10), penile torsion (n=7), hypospadias (n=2), meatal stenosis (n=1) were detected in 29 patients (29/318, 9%). The mean age was 4 years (2 months - 9 years) in this group of patients. The cases with penile anomalies are listed in Table-II.

**Table-II:** Patients with penile anomalies are listed.

Case	Age (year)	Penile Anomaly
1	3	rotation anomaly, chordee without hypospadias
2	8	penoscrotal web, glanular hypospadias
3	10	penoscrotal web
4	16 months	penoscrotal web
5	2	rotation anomaly, penoscrotal web
6	8 months	penoscrotal web
7	2	chordee without hypospadias
8	3	chordee without hypospadias
9	6	chordee without hypospadias
10	7	chordee without hypospadias
11	8	penoscrotal web
12	4	rotation anomaly
13	2	glanular hypospadias
14	4	chordee without hypospadias
15	2 months	penoscrotal web
16	3	chordee without hypospadias
17	8	penoscrotal web
18	1	meatal stenosis
19	4	penoscrotal web
20	2	penoscrotal web
21	7	penoscrotal web
22	1	rotation anomaly
23	2	penoscrotal web
24	4	rotation anomaly
25	9	penoscrotal web
26	16 months	rotation anomaly, chordee without hypospadias
27	4 months	penoscrotal web
28	9	rotation anomaly, chordee without hypospadias
29	6	chordee without hypospadias

**Fig.1:**
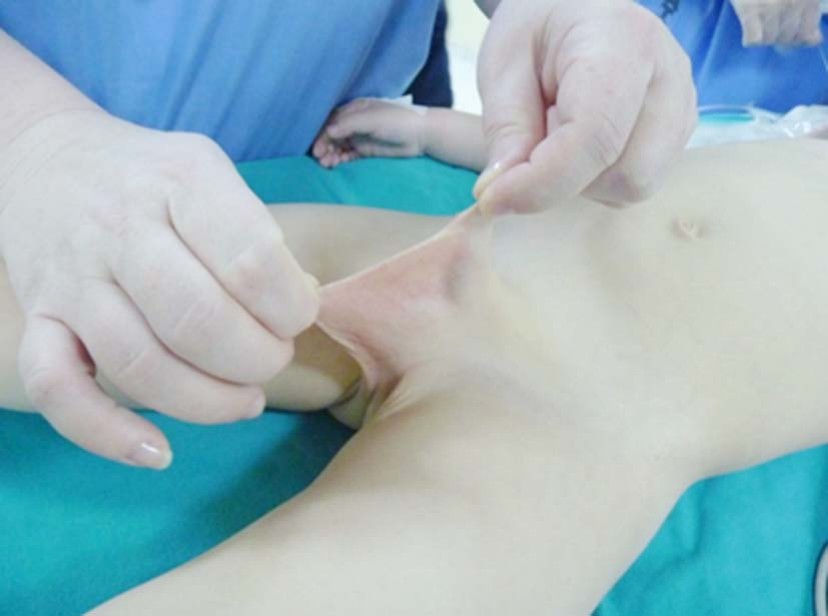
Webbed penis with a distorted penoscrotal angle.

**Fig.2:**
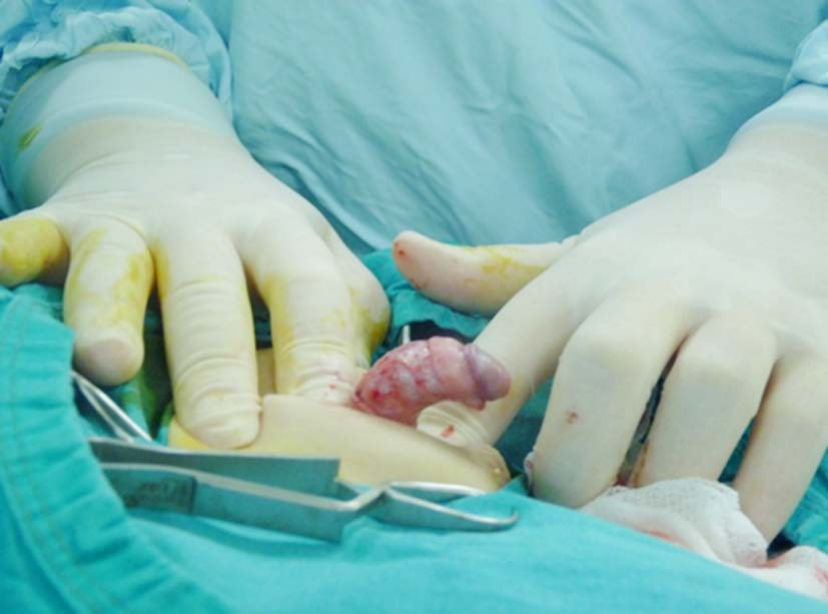
Chordee without hypospadias, the penis is degloved.

The web excision together with circumcision was performed in cases with webbed penis. Fig.1 shows a patient with a penoscrotal webbing. Penile degloving and excision of the fibrous bands over the penile shaft were sufficient to correct chordee in nine patients but dorsal plication was carried on in one boy. The ventral curvature in a patient is showed in Fig.2.

Minor bleeding developed in the postoperative period in four patients (4/318). All of them stopped with tighter dressing and did not require any surgical intervention. No infection was detected in this series. The swelling persisted longer in those patients with penile anomalies in whom penile degloving had to be performed.

## DISCUSSION

Circumcision is one of the most commonly applied surgical procedures in children, especially in those regions where it is ritually performed due to social and religion-based factors.^1^-^3^ Routine circumcision is commanded in Judaism and is accepted as sunnah in Islam. While it is generally carried on in the neonatal period in jews, there might be differences between the age distribution of boys undergoing circumcision in Islamic countries. Baky Fahmy et al. stated that nearly all of the routine circumcision is performed between 1 day to 7 weeks of age in Egypt.^3^ The mean age at admission was found to be 6.5 years in the presented series. Though neonatal circumcision is also becoming popular in bigger cities in Turkey, most of the parents still want their children to be aware of the process and also the celebrations they organize so they generally postpone the procedure by a few years. The higher incidence of admission in June can be explained with the initiation of summer vacation of schools in Turkey which is generally accepted to be the most suitable period for the operation.

The main indications for circumcision are recurrent balanoposthitis due to pathologic phimosis and recurrent urinary tract infections besides the social and religious reasons.^1^,^4^,^5^ Normal foreskin is non retractable at birth and is referred as “physiologic phimosis”. Physiologic phimosis resolves and the preputium becomes retractable by the age of 3 in almost 90% of boys.^4^,^6^,^7^ Many boys with physiologic phimosis are referred to pediatric surgeons even after forcefull trials of retraction. Ballooning in the distal penis might be observed due to physiologic phimosis but it is also not an indication for circumcision. The medical and nursing practitioners should be enlightened about the natural progression of the foreskin development in order to prevent admissions of the frightened parents in expectance of urgent operation. Pathologic phimosis, on the other hand, can cause recurrent balanoposthitis attacks as it was detected in 39 cases of this series. The children with prenatally detected urinary tract anomalies such as hydronephrosis or vesicoureteric reflux are more vulnerable to urinary tract infections and circumcision is generally recommended in the follow-up of these children. Zareba et al. declared that uncircumcised status of a boy was one of those independent risk factors for febrile urinary tract infection.^2^,^5^,^8^,^9^

Circumcision is a frequently performed procedure as the Islamic population approaches to 98% of our country. The boys were mainly circumcised in non-medical settings by some people traditionally called “circumciser”, who generally did not have a medical training prior to the recent arrangements by the Ministry of Health. Nowadays it is only permitted in a hospital setting. But the surgeons still have to face with the parents underestimating the procedure. Our recommendation is not to perform circumcision between 2-5 years of age if there is not any medical indication. The child who becomes aware of the developing genitalia, is more vulnerable pyschologically in this period of gender identity formation. General anesthesia is recommended as it is more convenient both for the patient and the surgeon.

**Fig.3:**
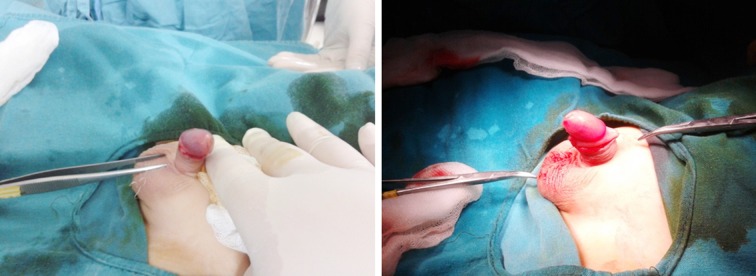
(a) The penis is found to be rotated to counterclockwise direction. (b) Penile torsion is corrected.

The most important advantage of admission to a hospital for circumcision is that the physical examination of the penoscrotal area by a surgical practitioner creates an opportunity to detect any hidden abnormality and if needed the child can be referred to an expert dealing with the surgical reconstruction of the genital anomalies. There is a wide spectrum of anomalies in the penoscrotal area.^3^-^6^ Different type of penile anomalies were detected in 9% of the circumcised boys in this series of patients. The penoscrotal angle is distorted in cases with webbed penis.^6^,^10^ Penoscrotal webbing was the most common accompanying anomaly in the presented series. Baldinger et al. stated that the penile torsion is observed in 2-27% of the newborns and added that there will not be any functional or physiologic significance unless the torsion is > 90°. The incidence of penile torsion was found to be 2% in the presented study and all of them were rotated counterclockwise (Fig. 3a and 3b). The torsion was corrected during circumcision.^4^,^6^

**Fig.4:**
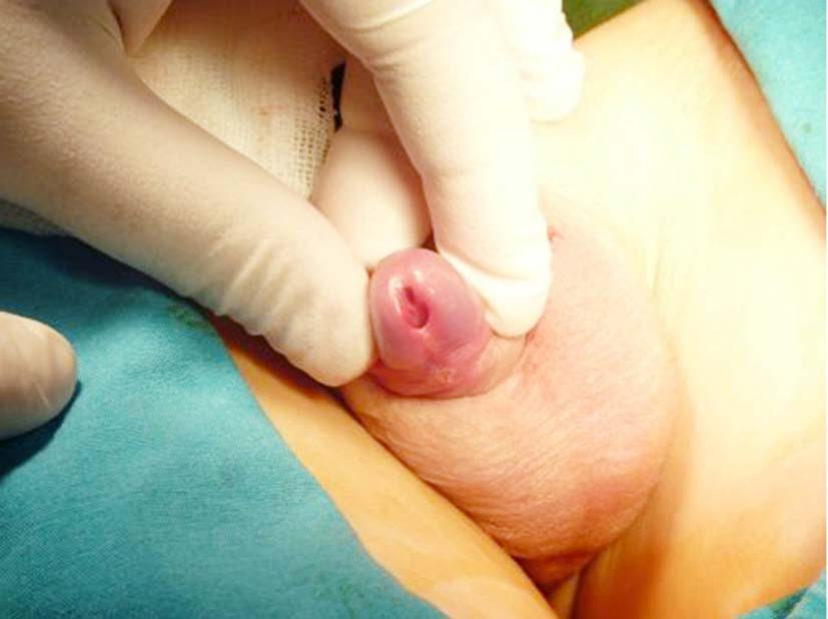
Glanular hypospadias is detected with the retraction of the foreskin.

Chordee generally accompanies hypospadias but chordee without hypospadias is observed more common than it is suspected. The structural anomalies of dartos are accused for its development but Wan et al also included skin tethering, corpora cavernosa disproportion to fibrotic dartos and Buck fascia as factors causing ventral penile curvature.^4^,^11^ Chordee without hypospadias was detected in 3% of boys of our study and only one case necessitated dorsal plication. Chordee can cause pyschosocial problems, painful intercourse and deviations in the urinary stream while standing. Polat et al. concluded that early surgical correction is possible as tunica albuginea plication was also effective in prepubertal period.^6^,^12^ The two glanular hypospadias cases, one is showed in Fig.4, were detected when the foreskin was retracted during circumcision. Meatoplasty was performed in one and MAGPI was carried in the second case.

In conclusion admission to a hospital for circumcision provides an opportunity to examine the genitalia of the boys properly. The different types of penile anomalies can be detected at that time of physical examination and appropriate correction can be performed together with circumcision. But the most important goal is to change the public perception of circumcision who generally underestimates the procedure. They should be informed about all the complications and consequences in order to have their children operated in a hospital setting by the medical staff.
